# A Rare Concurrent Presentation of Typhoid Fever with Bacteremia and Pulmonary Tuberculosis

**DOI:** 10.26502/acbr.50170456

**Published:** 2025-05-16

**Authors:** Shuhua Guo, Minghsun Liu

**Affiliations:** 1 Southern California Hospital Culver City, Culver City, California; Coast Plaza Hospital, Norwalk, California, USA; 2 Infectious Diseases, Centinela Hospital Medical Center/PrimeWest Internal Medicine Program, Inglewood, California; Woundtech, Hollywood, Florida, USA

**Keywords:** Typhoid fever, tuberculosis

## Abstract

This case report describes an unusual presentation of concurrent typhoid fever and pulmonary tuberculosis (TB) in an 82-year-old female. She was admitted with syncope and dehydration and initially suspected of having pneumonia and a urinary tract infection (UTI). Urine and blood cultures rapidly identified gram-negative rods, while CT imaging showed cavitary lesions in the bilateral upper lobes and left lower lobe. The gram-negative rods were identified as *Salmonella enterica subspecies enterica Typhi*, and sputum acid-fast bacilli (AFB) PCR and cultures confirmed the presence of *Mycobacterium tuberculosis* complex. The patient was treated for both typhoid fever and pulmonary tuberculosis. This rare case highlights the clinical challenge of distinguishing whether a single disease process is responsible for multiple symptoms (Occam’s Razor) or if multiple diseases are concurrently affecting the patient (Hickam’s dictum). Here, two distinct infections explained the complex presentation. Although typhoid fever was diagnosed first and could rarely be associated with pulmonary abscesses, cavitary lung lesions are more commonly seen in pulmonary tuberculosis. This case underscores the importance of considering multiple concurrent infections in complex clinical scenarios.

## Case Report

An 82-year-old female with a medical history of hypertension, type 2 diabetes mellitus, and hyperlipidemia presented to the emergency department following a syncopal episode that resulted in an occipital laceration. She reported experiencing episodes of dark, green, malodorous stools and dizziness. On admission, her vital signs included a temperature of 99.7°F, heart rate of 114 bpm, and blood pressure of 104/76 mmHg. Initial laboratory tests showed a white blood cell (WBC) count of 9.6 × 10^9/L with a left shift. A COVID-19 test was negative, and urinalysis was positive for pyuria. Stool culture and Clostridium difficile antigen tests were both negative. Chest X-ray suggested asymmetric pneumonia. Blood and urine cultures were obtained, and she was admitted for syncope and dehydration, receiving intravenous ceftriaxone and azithromycin for presumed pneumonia and urinary tract infection (UTI). Her initial labs otherwise were notable for low hemoglobin and hematocrit (8.2 gm/dL/26.6%) and hyperglycemia (239 mg/dL).

The patient, originally from Korea, immigrated to the U.S. over 30 years ago and lives at home with one of her sons. She is retired, with no recent travel history and no known sick contacts or previous history of positive PPD or Quantiferon TB Gold tests.

On the second hospital day, her WBC count increased to 27 × 10^9^/L and she experienced acute atrial fibrillation with rapid ventricular response and hypotension. She subsequently required norepinephrine for hypotension. Infectious disease consultation was requested to manage likely septic shock. Urine culture was growing gram-negative rods (GNR). Ceftriaxone was switched to cefepime empirically. CT of chest, abdomen, and pelvis without contrast was ordered to better characterize the source of her sepsis.

CT imaging of the chest, abdomen, and pelvis revealed bilateral upper lobe and left lower lobe cavitations, bilateral pleural effusions, and a left mid to lower pole renal staghorn calculus with mild left-sided hydroureteronephrosis extending to the ureteropelvic junction. The CT abdomen also showed colonic diverticulosis without evidence of enteritis or colitis.

By hospital day 3, blood culture was also growing GNR in both sets. Concerns about pulmonary tuberculosis arose due to cavitary lesions on imaging, leading to the implementation of airborne precautions and collection of sputum for acid-fast bacilli (AFB) PCR and smear with culture. Galactomannan, Coccidioides immunodiffusion and 4th Generation HIV test were also ordered and sent. Antibiotic regimen was escalated to include meropenem IV and vancomycin IV empirically.

By hospital day 4, the GNR from urine culture was identified as *Salmonella* species. Based on culture results and imaging findings, a high suspicion of extraintestinal *Salmonella* involving bacteremia, visceral lung infection, and descending urinary tract infection was the main working hypothesis.

Much to our surprise, AFB smears were positive for AFB organisms and PCR was positive for *Mycobacterium tuberculosis* complex. The Quantiferon Gold TB test was positive. In addition to ceftriaxone, the patient was started on rifampin, isoniazid, pyrazinamide, and ethambutol (RIPE) plus pyridoxine 50 mg oral daily. An outpatient eye examination was requested. HIV 4th Generation Test, stool culture, galactomannan, and Coccidioides immunodiffusion were all negative.

The patient was treated with ceftriaxone 2 gram IV daily for 4 weeks for severe extraintestinal Salmonellosis. During the patient’s treatment course, we did not know the exact identification of the Salmonella isolate. Public health did ultimately identify the isolate as *Salmonella typhi*. Repeat urine culture was negative. Blood culture clearance occurred approximately 48 hours after initiation of ceftriaxone. Repeat AFB sputum smear was negative after three weeks of RIPE therapy, consistent with TB treatment response. Patient’s hospital course totaled 28 days. Patient was eventually discharged home on RIPE therapy plus pyridoxine with public health follow-up.

## Discussion

This case underscores a complex and rare presentation that illustrates the essential need for heightened awareness of overlapping infections in elderly or immunocompromised patients. The combination of typhoid fever with *Salmonella* bacteremia plus UTI and pulmonary TB challenges clinicians to maintain a high index of suspicion and apply robust infection control and comprehensive treatment approaches.

In this case, the work-up for pulmonary TB was started after finding cavitary lung lesions on chest CT. After finding *Salmonella* species in blood and urine cultures and given reports of *Salmonella*-induced pulmonary abscess [1], we had preliminarily concluded that all the clinical findings are due to severe, extraintestinal Salmonellosis. When pulmonary TB was also ruled in, it was certainly a case of Hickam’s dictum rather than Occam’s razor [2].

For most of the hospitalization, we did not know if the patient had typhoid fever or extraintestinal *Salmonella* infection. Extraintestinal *Salmonella* infections are serious complications of *Salmonella* gastroenteritis, encompassing bacteremia, endovascular infection, endocarditis, musculoskeletal infection, visceral infection, and urinary tract infection [3–6]. bacteremia poses severe risks, including seeding to organs such as the lungs and urinary tract, as seen here [7]. Risk factors include age >50 years, immunocompromised states (e.g., HIV, transplant recipients, chronic corticosteroid or immunosuppressive therapy, sickle cell disease, cancer undergoing chemotherapy). The pathophysiology involves altered colonic permeability and bacterial translocation. Presentations range from febrile illness to septic shock. Primary bacteremia (bacteremia not associated with recent gastroenteritis) suggests an underlying immune defect or recrudescent infection [8]. Diagnosis is confirmed via blood culture.

In contrast, typhoid fever, caused by *Salmonella enterica* serotype *Typhi* or *Paratyphi*, presents with a more insidious onset of prolonged fever, abdominal pain, and, in some cases, a characteristic rash known as “rose spots” [9,10]. Unlike nontyphoidal Salmonella, typhoid fever is primarily a systemic disease from the outset, often without preceding diarrhea. It requires prompt antibiotic treatment to prevent complications such as intestinal perforation or severe systemic illness.

Even though the pulmonary findings of cavitary lesions turned out not to be related to the patient’s Salmonellosis, her presentation remains unique in that her abdominal CT reports presence of a staghorn calculus. Staghorn calculus is associated with *Proteus mirabilis* or other urease-producing bacterial species whereas *S. typhi* is urease negative [9]. The direct association of the *S. typhi* isolate with the staghorn calculus on CT remains unclear. However concurrent presence of urolithiasis and *Salmonella* in urine culture has been reported [11].

First-line treatment for typhoid fever includes third-generation cephalosporins (ceftriaxone or cefotaxime) or fluoroquinolones (ciprofloxacin or levofloxacin). Azithromycin (for uncomplicated illness) or carbapenem (for complicated illness) should be considered for patients with travel history to Iraq or Pakistan [12]. Immunocompetent patients require a 14-day antimicrobial course, while both immunocompetent and immunocompromised patients usually need close follow-up for complications or recrudescence [10,13].

## Conclusion

This case serves as a critical reminder for clinicians of the need to balance Occam’s Razor with Hickam’s Dictum when diagnosing complex medical cases. While the simplest explanation is often sought, multiple coexisting conditions can and do occur, especially in vulnerable populations like the elderly and immunocompromised. Clinicians must be adept at identifying and managing multiple infections simultaneously, using comprehensive diagnostic and treatment strategies to prevent disease progression and reduce mortality. This approach not only expands our understanding of unusual infectious presentations but also reinforces the necessity of a multidisciplinary perspective in patient care.
